# Screening the dysfunctional grief and its associated factors due to a death from covid-19 in Tunisia

**DOI:** 10.1192/j.eurpsy.2024.1043

**Published:** 2024-08-27

**Authors:** F. Askri, M. Dhemaid, K. Mahfoudh, S. Jedda, U. Ouali, A. Aissa

**Affiliations:** PSYCHIATRY A, RAZI HOSPITAL, Manouba, Tunisia

## Abstract

**Introduction:**

In Tunisia, the 2019 corona virus pandemic was a challenging health situation, with more than 28 000 confirmed deaths in May 2022. The pandemic was responsible for people losing their beloved ones in a sudden and brutal ways. Even though the numbers of bereaved people had been escalating, little attention was paid toward their mental health. Grief is a normal response to losing someone close. However, recent studies have shown that the covid-19 grief is more severe than other causes of grief. It not only causes a negative impact on the bereaved life aspects but also creates severe consequences in the society. Screening a possible dysfunctional grief is a major need to prevent serious outcomes.

**Objectives:**

To identify the prevalence of covid-19 dysfunctional grief and find out the possible associated risk factors to it.

**Methods:**

A cross sectional online survey designed using Google Forms and distributed on social media platforms (Facebook, Instagram, WhatsApp) was conducted from 16 February 2022 to 05 May 2022. The participants provided information related to socio-demographic data. Covid-19 grief scale was assessed using the pandemic grief scale, which was translated into Arabic but not validated.

**Results:**

A sample of 106 participants were recruited to this study .The sample was composed of Approximately 72% female and 28 % males, most of them were aged between 26 and 35 years old (37.7% ) . Overall, individuals who lost a loved one more than 06 months period were more frequent (81%). 91.7 % of the sample scored above the cut score of 7 on the PGS.

Covid-19 grief was higher among those who sought psychological help (p = 0.02). In this sample, there was no associated risk factors between different socio-demographic characteristics and dysfunctional grief, as well as no correlation were found between period of time since the loss and dysfunctional grief ( rho = 0.186, p = 0.56) .

**Conclusions:**

Although our study did not find a significant high prevalence of dysfunctional grief giving the small number of participants. More studies and screening must be conducted to identify those at risk of developing dysfunctional grief to prevent the serious individual and general outcomes.

**Disclosure of Interest:**

None Declared

